# Factors associated with antenatal and delivery care in Sudan: analysis of the 2010 Sudan household survey

**DOI:** 10.1186/s12913-015-1128-1

**Published:** 2015-10-04

**Authors:** Muna Hassan Mustafa, Abdel Moniem Mukhtar

**Affiliations:** Faculty of Medicine, International University of Africa, Khartoum, 12223 Sudan; Department of Family and Community Medicine, Faculty of Medicine, King Abdulaziz University, Jeddah, Saudi Arabia; Research Office, Public Health Institute, Khartoum, Sudan

**Keywords:** Antenatal care, Place of delivery, Institutional delivery

## Abstract

**Background:**

Every day, globally approximately a thousand women and girls needlessly die as a result of complications during pregnancy, childbirth or the 6 weeks following delivery. The majority of maternal deaths are avoidable and could be prevented with proven interventions to prevent or manage complications during pregnancy and child birth. The aim of this study was to examine factors associated with underutilization of maternal health services in Sudan.

**Methods:**

Data was obtained from the Sudan Household Health Survey 2010(SHHS). The SHHS collected data from 5730 women, aged 15–49 years and who were pregnant in the last 2 years preceding the survey. The selection of the respondents was through a multi-stage cluster sampling technique. Interviews were conducted with respondents to collect data about their demographic characteristics, reproductive history, pregnancy and child delivery. Univariate analysis and logistic regression were used to analyze the data.

**Results:**

The factors associated with receiving antenatal care were, higher educational level (odds ratio (OR) = 3.428, 95 % CI 2.473–4.751 – *p* value 0.001), higher household wealth (OR 1.656, 95 % CI: 1.484–1.855 – *p* value 0.001) and low parity (OR =1.214, 95 % CI: 1.035–1.423 – *p* value 0.017). The factors associated with institutional delivery were higher educational level (OR = 1.929, 95 % CI: 1.380–2.697 – *p* value 0.001), high household wealth (OR = 2.293, 95 % CI: 1.988–2.644 *p* value 0.001), urban residence (OR = 1.364, 95 % CI: 1.081–1.721 *p* value 0.009), low parity (OR = 2.222, 95 % CI: 1/786–2.765 *p* value 0.001), receiving ANC (OR = 3.342, 95 % CI: 2.306–4.844 *p* value 0.001) and complications during pregnancy (OR = 1.606, 95 % CI: 1.319–1.957 *p* value 0.001).

**Conclusions:**

The factors associated with both antenatal care use and institutional delivery are similar and interventions to target these include expanding female education and improving coverage and affordability of health services.

## Background

Although pregnancy and childbirth are natural and usual processes, they can put women at risk of complications. According to the World Health Organization (WHO), around a thousand women needlessly die every day as a result of complications during pregnancy, childbirth or the 6 weeks following delivery. Almost all (99 %) of these deaths occur in developing countries [1–3]. In addition, acute morbidity may affect over 50 million pregnancies/deliveries, and severe chronic and long-term disabilities, such as fistulas and prolapse, affect an estimated 10 million women each year [3–7].

In Sudan, the levels of maternal mortality and morbidity are among the highest in the Eastern Mediterranean region and globally. The second round of the Sudan Household Health Survey (SHHS-2) conducted in 2010, found there were 215.6 maternal deaths for every 100,000 live births [8].

The causes of maternal death are remarkably consistent across the developing world. Direct obstetric complications (e.g., post-partum haemorrhage, puerperal sepsis, pre-eclampsia and eclampsia, obstructed labour and abortion) account for 80 % of these deaths, with indirect causes such as malaria, anaemia and HIV/AIDS accounting for the remaining 20 % [4–6].

It has been estimated that 88–98 % of maternal deaths are avoidable [5] and could have been prevented, with proven interventions to prevent or manage complications becoming increasingly well-known [3] and considered central to improved maternal health [5, 9].

Antenatal care (ANC) is an effective health intervention for preventing maternal morbidity and mortality [6]. The antenatal period represents an important opportunity for identifying potential risks during pregnancy or at delivery, therefore enabling the necessary prompt management. Through ANC services, women receive assistance in developing a birth plan, and become more prepared for parenting after childbirth [4, 10]. ANC also provides opportunity to inform women about danger signs and symptoms for which immediate assistance should be sought from a health care provider [2].

Access to care from a trained health care worker during delivery is crucial in reducing maternal deaths. A skilled health professional can manage normal deliveries, administer interventions to prevent and manage life-threatening complications and refer the patient to a higher level of care when needed [3, 5, 9].

Skilled attendance at delivery is advocated as the single most important factor in preventing maternal deaths [3]. Skilled attendants can perform deliveries either at home or in a health facility. However, it has been argued that the most efficient application of skilled attendants in lower income countries is to place them in health centres with referral capacity. In practice, skilled attendance in most countries is synonymous with delivery at a facility [3].

Undesirable outcomes of home delivery including high maternal and perinatal mortality have been well-documented in developing countries. Studies from different developing countries have shown high rates of mortality and obstetric complications associated with home births that took place without a trained attendant [7].

As in other developing countries, ANC services in Sudan are underused. The first round of the Sudan Household Health Survey (SHHS-1) conducted in 2006 found that 69.6 % of pregnant women attended ANC at least once during their pregnancy [11]. The SHHS-2, conducted in 2010, reported a slight increase to 74.3 %, although this remains lower than the national target of 90 % of pregnant women accessing ANC [12].

The proportion of women delivering at a health facility increased slightly from 19.4 % in 2006, achieving the national target of 20 % of all deliveries at health facilities by 2010 [8, 11]. However, the proportion of health facility deliveries is low, and poses a challenge to achieving the Millennium Development Goals (MDGs).

Many initiatives have been implemented to increase coverage of maternal health services. These include the expansion of maternal and newborn health services across Sudan through the renovation and construction of health facilities, and building the capacity of health care providers [12]. However, the presence of ANC and delivery services does not guarantee they will be used by the target population. Underuse of modern health care services is a major reason for poor health in developing countries [10]. The proportion of women who attend the recommended number of ANC visits in developing countries remains low, although there has been an increase from 35 % in 1990 to 51 % in 2009 [9]. However, the proportion of deliveries attended by skilled health personnel has not increased from 1990, remaining at 65 % in 2009 [9].

Many factors have been found to affect the demand and use of ANC and delivery services, including sociodemographic characteristics, economic status, autonomy and obstetric history [2].

To promote the uptake of maternal health services in Sudan, it is essential to identify factors that should be considered. This will inform the development of context-specific strategies and interventions, and may promote the use of such services, ultimately leading to a reduction in maternal morbidity and mortality in Sudan.

The present study aimed to examine factors associated with the use of ANC and institutional delivery in Sudan.

## Methods

### Study design and setting

The SHHS is a cross-sectional, national survey first conducted in 2006. It covers all of the states of Sudan. This survey provided information on key household, children and women health indicators.

### Data source

The SHHS-2 was conducted in 2010. It represents a major tool that generated data to assess the situation of children and women in Sudan, and to monitor progress towards selected national development goals and MDGs. The methodology and content of the SHHS-2 are based on the models and standards developed by the global Multiple Indicator Cluster Survey (MICS) project, of UNICEF, and also on the modules of the Family Health Survey of the Pan Arab Project for Family Health (PAPFAM). The survey was national, and covered all of the current 18 states of Sudan.

The survey used a two-stage, cluster sampling design. In each state, 40 clusters were selected, with 25 households selected from each cluster. In the selected households, data were collected for all women aged 15–49 years; men aged 15–49 years; and children below 5 years of age. The present analysis used data collected for women. All women were interviewed by female interviewers using a questionnaire developed specifically for women. This questionnaire covered demographic characteristics, reproductive history, pregnancy, antenatal and postnatal care, assistance at delivery, tetanus immunisation and family planning.

### Study population

Women aged 15–49 years who were pregnant in the two years before the SHHS-2 (*n* = 5730).

### Variables

The primary outcomes of the present study were: receiving ANC services (yes, no) and the place of delivery (home, institution/health facility).

Two conceptual frameworks were adapted from Andersen’s behavioural model framework [13] for use of health services, to group factors potentially associated with receiving ANC services and place of delivery. Full adaptation of Andersen’s model was not possible because of the unavailability of data on some of the original framework factors.

**Fig. 1:**
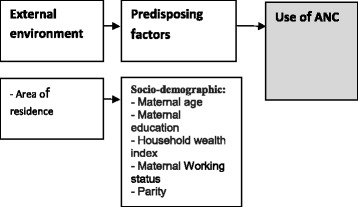
Theoretical framework of factors associated with use of antenatal care services in Sudan (adapted from Andersen behavioural model [13])

**Fig. 2:**
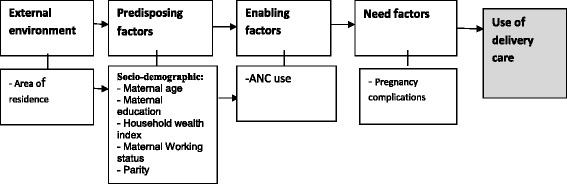
Theoretical framework of factors associated with institutional delivery in Sudan (adapted from Andersen behavioural model [13])

Six potential factors associated with receiving ANC (Fig. 1) and eight factors associated with place of delivery (Fig. 2) were identified and categorised into four main groups: external environment, predisposing, enabling and need factors. For receiving ANC and place of delivery, the external environment was represented by the area of residence (urban, rural). The variables of the predisposing factors for receiving ANC and place of delivery included: 1) Age, categorised as younger and older age groups with the median age (35 years) taken as the cut-off point; 2) wealth index, estimated from a combination of variables and categorised into five classes (from poorest to richest) for univariate analysis. For the multivariate logistic regression model, wealth index was taken as a continuous variable; 3) educational level, categorised into four levels (none, primary, secondary and above, and adult/informal education); and, 4) parity, categorised as a dichotomous variable (1–2, more than two) for univariate analysis and a continuous variable for the multivariate logistic regression model. The enabling factors included in the framework were only for place of delivery (Fig. 2), and were represented by receiving ANC during the index pregnancy, which were categorised as ‘yes’ or ‘no’. Need-related factors were also only found for the place of delivery, and were represented by occurrence of complications during the index pregnancy. Originally, the variable had eight types of pregnancy related complications; after discussion, these were categorised as either serious complications (severe bleeding, convulsions, hypertension, jaundice, urethral discharge with fever), or no/non-serious complications (fever, lower abdominal/back pain, and burning urination).

### Statistical analysis

Frequency tables were generated to describe participant characteristics stratified by different independent variables. Contingency tables were constructed, and a Chi-square test was used to examine factors associated with receiving ANC and place of delivery. A p value of less than 0.05 was considered significant.

Variables found to be significantly associated with the two study outcomes on at least one category were entered into a multivariate logistic regression model, to evaluate the effect of the independent variables on receiving ANC and place of delivery. All statistical analyses were performed using SPSS Version 18 (PASW Statistics for Windows, Version 18.0. Chicago: SPSS Inc.).

### Ethics

Ethical permission for the SHHS-2 was obtained from the National Ethical Review Committee, Federal Ministry of Health, Khartoum, Sudan. Informed verbal consent was obtained from all women aged 15–49 years before data collection. In case of women below 18 years of age, the consent was obtained from their parents or guardians.

Participation was voluntary. Privacy was assured for all women during interviews.

The data set supporting the results of the present study were obtained with permission from the Central Bureau of Statistics, Khartoum Sudan (www.cbs.gov.sd).

## Results

Table 1 presents the baseline characteristics of women included in the present analysis. Table 2 shows factors associated with receiving ANC. Mothers from urban areas were more likely to receive ANC services compared with mothers from rural areas (urban 87.6 %, rural 74.3 %; *p* = 0.001). Women in the younger age group (15–34 years) were more likely to receive ANC services compared with women in the older age group (35–49 years) (younger group 78.5 %, older group 74.2 %; *p* = 0.001).

**Table 1:** Characteristics of women aged 15–49 years who gave birth in Sudan from 2008–2010

Background characteristics	No.	Percent
Area of residence-Missing: 389 (6.4 %)		
Rural	4134	72.8
Urban	1541	27.2
Maternal age-Missing: 389 (6.7 %)		
Younger age group (15–34 years)	4317	76.1
Older age group (35–49 years)	1358	23.9
Wealth index quintiles-Missing: 390 (6.4 %)		
Poorest	1319	23.2
Second	1255	22.1
Middle	1263	22.3
Fourth	1065	18.8
Richest	772	13.6
Education-Missing: 389 (6.4 %)		
None	2522	44.4
Primary	1917	33.8
Secondary +	961	16.9
Adult education/informal education	275	4.8
Working status-Missing: 488 (8.1 %)		
Not working	5232	93.8
Working	344	6.2
Parity (number of pregnancies)-Missing: 390 (6.4 %)		
> 2	3681	64.9
1–2	1993	35.1
Received ANC-Missing: 94 (1.7 %)		
Yes	4326	77.4
No	1260	22.6
Complications during pregnancy-Missing: 2419 (42.6 %)		
No/Non serious complication	1752	53.7
Serious complication	1509	46.3

**Table 2:** Factors associated with antenatal care use in Sudan, 2010

*n = 6064*	Received ANC		*P*- Value
	Yes	No	
External environment			
Area of residence-Missing: 389 (6.4 %)			
Rural	3053 (73.9 %)	1081 (26.1 %)	0.001
Urban	1346 (87.3 %)	195 (12.7 %)	
Predisposing factors			
Maternal age-Missing: 389 (6.7 %)			
Younger age group (15–34 years)	3391 (78.5 %)	926 (21.5 %)	0.001
Older age group (35–49 years)	1008 (74.2 %)	350 (25.8 %)	
Education- Missing: 389 (6.4 %)			
None	1692 (67.1 %)	830 (32.9 %)	0.001
Primary	1626 (84.8 %)	291 (15.2 %)	
Secondary +	904 (94.1 %)	57 (5.9 %)	
Adult education/informal education	177 (64.4 %)	98 (35.6 %)	
Wealth index quintiles-Missing: 390 (6.4 %)			
Poorest	880 (66.7 %)	439 (33.3 %)	0.001
Second	900 (71.7 %)	355 (28.3 %)	
Middle	942 (74.6 %)	321 (25.4 %)	
Fourth	939 (88.2 %)	126 (11.8 %)	
Richest	738 (95.6 %)	34 (4.4 %)	
Working status-Missing: 488 (8.1 %)			
Not working	4049 (77.4 %)	1183 (22.6 %)	0.001
Working	292 (84.9 %)	52 (15.1 %)	
Parity-Missing: 390 (6.4 %)			
> 2	2753 (74.8 %)	928 (25.2 %)	0.001
1–2	1646 (82.6 %)	347 (17.4 %)	

The probability of attending ANC services increased significantly for mothers with high educational attainment (secondary education and above 94.1 %, no education 67.1 %; *p* = 0.001), and for households with a higher wealth index (richest household 95.6 %, poorest household 66.7 %; *p* = 0.001). Women who were working were more likely to attend ANC than those who did not work (working 84.9 %, not working 77.4 %; *p* = 0.001). Another significant demographic factor identified was parity. Mothers of high birth rank children were more likely not to attend ANC (low rank 82.6 %, high rank 74.8 %; *p* = 0.001).

Table 3 presents factors associated with place of delivery. Women residing in urban areas were more likely to have an institutional delivery than women residing in rural areas (urban 39.7 %, rural 14.6 %; *p* = 0.001).

**Table 3:** Factors associated of place of delivery in Sudan, 2010

*n* = 5680	Place of delivery		*P*- Value
	Home delivery	Institutional delivery	
External environment			
Area of residence-Missing: 389 (6.4 %)			
Rural	3501 (85.4 %)	600 (14.6 %)	0.001
Urban	895 (60.3 %)	590 (39.7 %)	
Predisposing factors			
Maternal age-Missing: 389 (6.7 %)			
Younger age group	3363 (78.9 %)	898 (21.1 %)	0.462
Older age group	1034 (78 %)	292 (22 %)	
Education-Missing: 389 (6.4 %)			
None	2282 (91.2 %)	220 (8.8 %)	0.001
Primary	1419 (74.6 %)	482 (25.4 %)	
Secondary +	448 (49.2 %)	463 (50.8 %)	
Adult/informal education	247 (90.5 %)	26 (9.5 %)	
Wealth index quintiles-Missing: 390 (6.4 %)			
Poorest	1253 (95.6 %)	57 (4.4 %)	0.001
Second	1126 (90.4 %)	119 (9.6 %)	
Middle	1043 (83 %)	214 (17 %)	
Fourth	683 (64.6 %)	374 (35.4 %)	
Richest	292 (40.6 %)	427 (59.4 %)	
Working status-Missing: 488 (8.1 %)			
Not working	4084 (79.3 %)	1069 (20.7 %)	0.001
Working	220 (65.5 %)	116 (34.5 %)	
Parity (number of pregnancies)-Missing: 390 (6.4 %)			
> 2	3056 (84.2 %)	572 (15.8 %)	0.001
1–2	1340 (68.4 %)	619 (31.6 %)	
Enabling factors			
Received ANC-Missing: 94 (1.7 %)			
Yes	3200 (74 %)	1126 (26 %)	0.001
No	1196 (94.9 %)	64 (5.1 %)	
Need factors			
Complications during pregnancy-Missing: 2419 (42.6 %)			
No/non-serious complication	1425 (81.3 %)	327 (18.7 %)	0.001
Serious complication	1147 (76 %)	362 (24 %)	

In terms of sociodemographic factors, the results showed no statistically significant effect of age on place of delivery (*p* = 0.462). The probability of having an institutional delivery increased significantly among women with high educational attainment (secondary and above 50.8 %, no education 8.8 %; *p* = 0.001). The probability of having an institutional delivery also increased as the wealth index increased (richest 59.4 %, poorest 4.4 %; *p* = 0.001). Women who worked were more likely to have an institutional delivery compared with those who did not work (working 34.5 %, not working 20.7 %; *p* = 0.000). Parity was a further demographic factor that determined the place of delivery, with mothers of low birth rank infants (low rank 31.6 %) more likely to have an institutional delivery than mothers of high birth rank infants (high rank 15.5 %; *p* = 0.000).

In terms of enabling factors, women who received ANC services during pregnancy were more likely to have an institutional delivery when compared with those who did not (ANC 26 %, no ANC 5.5 %; *p* = 0.000).

The presence of serious complications during pregnancy (need factors) had a significant effect on the place of delivery. Women who encountered serious complications during pregnancy were more likely to have an institutional delivery (serious complications 24 %, no/non-serious complications 18.7 %; *p* = 0.00).

The use of ANC services was associated with a number of factors. To assess the contribution of each factor to the overall variance while controlling for all other included factors, we conducted a multivariate logistic regression analysis. In the final multivariate model, education level was associated with attending ANC (odds ratio [OR] 3.428, 95 % confidence interval [CI] 2.473–4.751, *p* = 0.001) indicating a strong association between attending ANC and a high education level (secondary and above) compared with women with no education. High household wealth was associated with attending ANC (OR 1.656, 95 % CI 1.484–1.855, *p* = 0.001). Low parity was also associated with attending ANC (OR 1.214, 95 % CI 1.035–1.423, *p* = 0.017), suggesting that low birth rank mothers are more likely to use ANC services compared with high birth rank mothers (Table 4). Multivariate logistic regression was conducted to assess factors associated with place of delivery. In the final multivariate model, urban residency was associated with institutional delivery (OR 1.364, 95 % CI 1.081–1.721, *p* = 0.009), indicating that those living in urban areas are more likely to have an institutional delivery than those living in rural areas. Mother’s education level (secondary and above) was associated with institutional delivery (OR 1.929, 95 % CI 1.380–2.697, *p* = 0.001), suggesting that women with higher education levels tend to choose an institutional delivery more than women with low or no education. High household wealth was associated with institutional delivery (OR 2.293, 95 % CI 1.988–2.644, *p* = 0.001), suggesting those of a higher wealth index tend to deliver more at institutions compared with mothers of a lower wealth index. Low parity was associated with institutional delivery (OR 2.222, 95 % CI 1/786–2.765, *p* = 0.001), indicating that women with low parity had more institutional deliveries than women with high parity. Attending ANC during the index pregnancy was associated with institutional delivery (OR 3.342, 95 % CI 2.306–4.844, *p* = 0.001), indicating that women who received ANC were more likely to have an institutional delivery for that pregnancy compared with those who did not. Encountering complications during pregnancy was also associated with institutional delivery (OR 1.606, 95 % CI 1.319–1.957, *p* = 0.001), suggesting that women who encountered serious complications during their pregnancy tended to deliver at institutions more than those who had no complications or non-serious complications during pregnancy (Table 5).

**Table 4:** Logistic regression analysis for variables associated with receiving antenatal care in Sudan, 2010

	Odds ratio	95 % confidence intervals		*p*-Value
		Lower	Upper	
Predisposing factors				
Age				
Younger age group (15–34 years)	1			
Older age group (35–49 years)	0.867	0.735	1.022	0.089
Education				
None	1			0.001
Primary	1.991	1.684	2.354	0.001
Secondary +	3.428	2.473	4.751	0.001
Adult education	0.783	0.598	1.023	0.073
Wealth Index				
Wealth index score	1.659	1.484	1.855	0.001
Parity				
Parity > 2	1			
Parity (1–2)	1.214	1.035	1.423	0.017

**Table. 5:** Logistic regression analysis for variables associated with place of delivery in Sudan 2010

	Odds ratio	95 % C.I. for odds ratio		*P* value
		Lower	Upper	
External environment				
Area of residence				
Rural	1			
Urban	1.364	1.081	1.721	0.009
Education				
Non	1			
Primary	1.666	1.297	2.141	0.001
Secondary +	1.929	1.380	2.697	0.001
Adult education	1.191	.688	2.063	0.533
Wealth index				
Wealth index(score)	2.293	1.988	2.644	0.001
Parity				
Parity >2	1			
Parity 1-2	2.222	1.786	2.765	0.001
Enabling factors				
Ante natal care				
No	1			
Yes	3.342	2.306	4.844	0.001
Need factors				
Complications during pregnancy				
No/Non-serious complication	1			
Serious complication	1.606	1.319	1.957	0.001

## Discussion

The present study demonstrated the association between a range of factors and attending ANC and place of delivery. Factors found to be associated with both attending ANC and institutional delivery were higher educational attainment, higher household wealth and low parity.

Our study highlighted an association between maternal education and attending ANC and institutional deliveries, findings that are consistent with the results of previous studies [2, 3, 5, 6, 10]. With few exceptions, all studies in this field found a strong and dose-dependent positive effect of educational level on use of maternal services [3].

Several pathways have been suggested through which maternal education might affect health-care use. For example, highly educated women are more likely to be aware about the importance of health services and more able to select the most appropriate service for their needs [3, 10]. In addition, educated women have more opportunities to enter into the labour market, and therefore have access to financial resources and more decision-making power [3, 10].

Although interventions targeting female education may not have a short-term effect on enhancing service use, education should still be considered as a key intervention to promote the use of ANC services, not only because it will increase women’s awareness, but it will also lead to increased empowerment of women and may improve their access to financial resources throughout their adulthood.

Our study showed that there was an increased likelihood of attending ANC and having an institutional delivery associated with increased household wealth. The role of household economic status on health service use has been reported in previous studies [3, 6, 7, 10], with women from households with a high wealth index being more likely to afford health services than those of a lower wealth index [10]. Although some of Sudan’s maternal health services are provided free of charge at government health facilities, additional costs of care-seeking are not covered, such as costs of transportation, supplies and the opportunity costs of travel and waiting time. This is especially relevant to institutional deliveries, where women are usually accompanied by other family members, further increasing care-seeking costs. Women from poor households may struggle to cover these additional costs, and are therefore less likely to seek maternal health services at an institutional level [3].

In contrast, home deliveries supervised by traditional birth attendants (TBAs) are usually perceived as affordable, as transportation costs are not necessary and payment is usually negotiable in terms of amount and timing, and can be in kind [3]. Economic status, when it becomes a determinant of health care use, implies access to health care is inequitable [13]. Coverage by health insurance to meet the cost of those services not provided free of charge, and expanding health services as near as possible to the target population are two measures that can be implemented to improve access for underprivileged populations and enhance maternal service use.

The association between high parity and low use of maternal health services observed in our study has been reported in previous studies [2, 10], which found that women with high parity tend to rely on their experience from previous pregnancies and do not feel the need for antenatal checks, believing they already know what to expect during pregnancy and childbirth. We observed a similar parity effect for the place of delivery, with our results showing that multi-parity was associated with home delivery. Other studies have reported similar results, with higher levels of service use reported for the first and lower order births when compared with higher order births [3, 7]. Unfortunate experiences in hospitals, quicker childbirth in multiparous women or having had an uncomplicated first delivery might explain why some multiparous women deliver at home. In contrast, as the first birth is known to be more difficult and a woman usually has no previous experience of delivery, they might be more likely to seek professional help and advice. In addition, women with several small children may experience greater difficulty in attending facilities for both ANC and child birth, because of the need to arrange child care [3, 7, 10].

Our findings emphasise the importance of raising the awareness among women about the risks associated with pregnancy and childbirth, and the importance of both ANC services and institutional delivery. Our findings also suggest that provision of family-friendly services as close as possible to the target population is necessary.

Our study also found that area of residence (urban or rural) was associated with institutional delivery, but not with ANC use. This may be explained by the inaccessibility of institutional delivery services in rural areas. In many rural areas in Sudan, primary health centres provide ANC but are not equipped for delivery services. In these areas, women depend on midwives to perform home deliveries. In areas where primary health centres are not available, midwives visit women at their homes to provide both ANC and child delivery services. Even when an institutional delivery is planned at a nearby urban area, additional challenges are presented by factors such as lack of or difficulty arranging transportation, especially if need arises at an inconvenient time.

In contrast, similar studies from other countries reported an advantage for women living in urban areas over women in rural areas with regard to ANC use, attributed to different service and social environments [2, 4, 10]. This inconsistency in results may indicate that in rural Sudan, free ANC services may lead to higher use of these services. This is despite the fact that in Sudan, urban women were found to have better educational attainment indicators than their rural counterparts [8]. This emphasises the importance of the availability of accessible, affordable health services in encouraging women to use ANC services.

The effect of the enabling factor (ANC use) was tested by the probability of having an institutional delivery. The results of the present study indicated that women who attended ANC services had an increased likelihood of having an institutional delivery. This is consistent with results of previous studies [5, 7, 10]. ANC is an opportunity for health workers to promote a specific place of delivery or give women information about the status of their pregnancy; which in turn informs their decisions on where to deliver. The association between ANC use and institutional delivery might be a reflection of the availability of and access to services, as women who reside closer to facilities are more likely to use ANC and delivery services [3]. In addition, ANC attendance can be a marker of familiarity in interacting with the health system and with the health facility [3, 5], with women who use ANC services being more likely to use facilities for delivery.

The presence of complications during the index pregnancy was included in our study to represent need factors. Our results showed that women who had encountered serious complications during their pregnancy were more likely to have an institutional delivery. This is consistent with the findings of previous studies in countries where deliveries attended by skilled personnel are low [5]. The factors associated with institutional delivery suggest that women opted for health facility delivery primarily when problems were encountered during pregnancy. The problems experienced during the index pregnancy might have made these women seek health services during pregnancy; with health workers subsequently recommending a health facility delivery [3].

This indicates that the complications encountered are translated into perceived need, and that women judged these complications to be of sufficient importance and magnitude to seek professional help. This in turn depends on women’s knowledge of danger signs and on their beliefs about the causes of these signs [13], again emphasising the importance of raising the awareness of women through appropriate community-based and intensive behavioural communication strategies. These strategies should reinforce women’s perception of danger signs, direct their decision-making towards appropriate action and finally, ensure that this knowledge will prompt the appropriate action [14, 15].

A major limitation of the present study was the lack of data on enabling factors such as the availability and accessibility of health services. The SHHS provided data related to service users, whereas data about the availability, accessibility and quality of health services were lacking. This made it difficult to test the effect of the full range of factors and their interactions.

Another limitation is the cross-sectional design of the present study, as it restricts the interpretation of the causality of factors associated with using maternal health services. Recall bias may be a further limitation, but its effect was minimised by including events occurring during the two years preceding the survey.

The main strength of the present study is the large, nationally representative sample which contributes to the validity of our results.

## Conclusions

In conclusion, we found that attending ANC was associated with higher maternal education, higher household wealth and low parity. Institutional delivery was associated with high maternal education, high household wealth, urban residency, low parity, attending ANC and history of pregnancy complications. Interventions to improve the use of maternal health services should include measures to promote female education, improve the coverage of services and increase the affordability of maternal health services.

## Abbreviations

ANC: Antenatal care
CI: Confidence interval
HIV/AIDS: Human immuno- deficiency virus/acquired immuno- deficiency syndrome
MDGs: Millennium developmental goals
MICS: Multiple indicator cluster survey
kOR: Odds ratio
PAPFAM: Pan arab project for family health
SHHS: Sudan household health survey
SPSS: Statistical package for social sciences
TBAs: Traditional birth attendants
UNICEF: United Nations children fund
WHO: World Health Organization
